# Isolated Ovarian Tuberculosis Mimicking Ovarian Carcinoma: Case Report and Literature Review

**DOI:** 10.4314/ajid.v5i1.66508

**Published:** 2011

**Authors:** SSEN Rabesalama, KL Mandeville, RA Raherison, HN Rakoto-Ratsimba

**Affiliations:** 1 Service de Chirurgie Viscérale, Centre Hospitalier Universitaire Joseph Ravoahangy Andrianavalona (CHU-JRA) Ampefiloha, BP 4150 Antananarivo 101 Madagascar; 2 Department of Infection and Population Health, University College London, Hampstead Campus, Royal Free Hospital, Rowland Hill Street, London, NW3 2PF; 3 Service d'Urologie, Centre Hospitalier Universitaire Joseph Ravoahangy Andrianavalona (CHU-JRA) Ampefiloha, BP 4150 Antananarivo 101 Madagascar

**Keywords:** Genitourinary tuberculosis, Ovarian tuberculosis, Ovarian carcinoma

## Abstract

Although genitourinary tuberculosis is common, reports of isolated ovarian tuberculosis are rare. However, its presentation can mimick that of an ovarian tumour, leading to diagnostic difficulties. A woman of 17 years presented with chronic pelvic pain, weight loss, a right ovarian mass on ultrasound, and a significantly elevated CA-125 level. A diagnosis of ovarian carcinoma was made, and laparotomy was performed with resection of the right ovary. Postoperative histological examination, however, revealed classic tuberculoid appearances, with no signs of malignancy. Antituberculosis treatment was commenced, with full resolution of her symptoms and a decrease in CA-125 level. Isolated ovarian tuberculosis is most common in young women living in endemic zones. CA-125 can be raised in both conditions, and imaging is rarely conclusive. Intraoperative frozen section of tissue specimens can be helpful if available. Early diagnosis of ovarian tuberculosis is vital as untreated disease can lead to infertility.

## Introduction

Tuberculosis (TB) remains a significant public health problem worldwide. There were an estimated 9.2 million new cases and 1.7 million deaths from TB in 2008 (Global Tuberculosis Control Report 2008). Although genito-urinary disease is common, isolated ovarian TB is rare (Nebhani et al, 2004). We report here a case in a young girl treated in Antananarivo, Madagascar. The clinical features and diagnosis of ovarian TB are discussed, with a review of the literature.

## Case Report

A nulliparous woman, aged 17 years, presented to hospital with a 2 month-history of pelvic pain. This was associated with a low-grade fever, weakness, and anorexia. She also reported a weight loss of 4kg in 6 months, with a BMI of just 15. She had received the Bacille Calmette-Guerin (BCG) vaccination at birth and there was no history of contact with tuberculosis.

Menarche was at age 12, with regular cycles, however her last menstrual period was over four months ago. Vaginal examination revealed a right lateral uterine mass tender to palpation. Blood tests showed a moderate anaemia with a haemoglobin of 10g/dL, and an erythrocyte sedimentation rate of 90mm. Tumour markers were measured, and the level of CA-125 was 450 units/mL (15 times the upper limit). HIV serology was negative.

**Figure 1:**
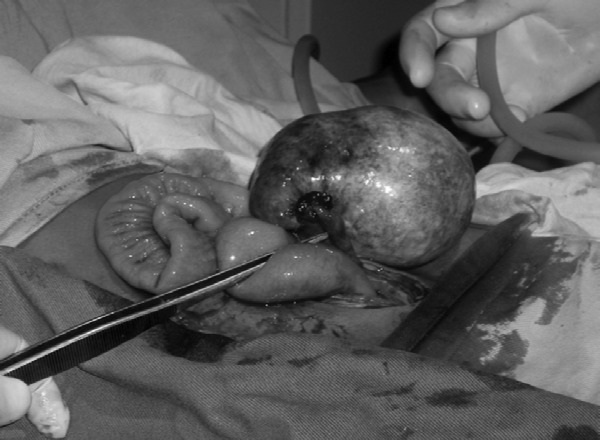
Intraoperative image of right ovarian mass measuring 60×45mm.

Plain radiography of chest and abdomen was normal. Pelvic ultrasound demonstrated a heterogenous right adnexal mass of 50 × 45mm and a small amount of ascites in the sac of Douglas. The initial diagnosis was ovarian carcinoma, and we proceeded to laparotomy. This revealed a discrete cystic mass of the right ovary which was fully excised (Figure 1). The rest of the peritoneal cavity was completely unremarkable.

**Figure 2:**
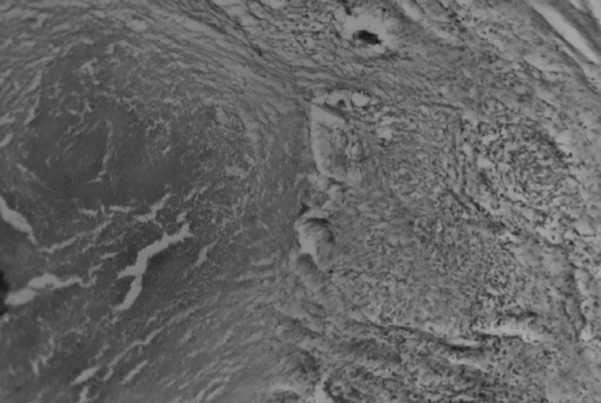
Histology slide showing caseous necrosis and giant cell proliferation, confirming ovarian tuberculosis (haematoxylin and eosin X100)

Postoperative histopathological examination showed giant cell proliferation with central caseous necrosis (Figure 2). There was no sign of malignancy, and the diagnosis was revised to ovarian tuberculosis. No other focus of TB was found, including pulmonary and urinary disease. Antituberculosis treatment was commenced, and continued for eight months as per current guidelines in Madagascar. Recovery was marked by complete resolution of the pelvic pain, a weight gain of 2 kg in 2 months, normalisation of her menstrual cycles, and a decrease in the CA-125 level.

## Discussion

Genito-urinary tuberculosis is the second most frequent location for extra-pulmonary tuberculosis, after the lymphatic system (Watfa and Michel, 2005). This site can represent up to 19% of gynaecological admissions in some developing countries (Sfar et al, 1990). The endometrium and fallopian tubes are almost always affected by the disease (Agarwal and Gupta, 1993). The ovaries were involved in 62.5% of cases in one study (Agarwal and Gupta, 1993). However, isolated ovarian TB with no other organ involvement as in this case, is rarely reported in the literature.

It classically affects young women aged 20–30 years who are living in endemic zones. However, with increased immigration, travel, and the re-emergence of tuberculosis worldwide, reports from Western countries are also found (Falk et al., 1980; Pesut and Stojsic, 2007, Straughn and Robertson,, 2000).

Pulmonary TB can be described prior to the ovarian disease, however this is not obligatory, as demonstrated by our case. Presenting symptoms include infertility, pelvic pain, abdmino-pelvic masses, ascites, weight loss and menstrual problems such as amenorrhoea and dysmenorrhoea (Nebhani et al, 2004). However, the patient can also be asymptomatic, which is estimated to account for at least 11% of cases in the population (Varma, 1991).

Pre-operative tests which may aid the diagnosis include a positive Mantoux (tuberculin) test, and staining for acid-fast bacilli in either ascitic or pleural fluid. However, these can be negative despite extensive disease (Jana and Dhali, 2007).

Ca-125 is an antigenic determinant which is expressed in most nonmucinous epithelial ovarian carcinomas, and is raised in more than 80% of cases (Nebhani et al 2004, Straughn and Robertson, 2000). It is very useful in postmenopausal women (Pesut and Stojsic 2007), where the positive predictive value for malignancy is nearly 95%. However, in premenopausal women, it can be elevated by benign conditions such as endometriosis, fibroids, and pelvic inflammatory disease, and indeed tuberculosis (Straughn and Robertson, 2000). In the case of ovarian TB, its level rarely rises above 500U/ml (450U/ml in this case). (Nebhani et al., 2004, Lantheaume et al 2003). Simsek et al.(1997) have shown that decreasing levels of CA-125 correlate with the resolution of the disease on antituberculous treatment. They suggest that serial measurements should be used to determine treatment efficacy.

Imaging has low specificity, with both an ovarian malignancy and a tuberculous abscess having similar appearances on ultrasound, competurised tomography, and magnetic resonance imaging (Lantheaume et al 2003). Both can be heterogenous masses, which can infiltrate omentum and neighbouring organs. Ascites and lympadenopathy are both frequently present, further confusing the diagnosis (Nebhani et al 2004). Ultrasound-guided transvaginal or transabdomenal biopsies may be used for preoperative diagnosis (Caspi et al 2000).

Laparoscopy has been a great advance as it allows the diagnosis of tuberculosis in more than 97% of cases whilst avoiding laparotomy (Nebhani et al 2004, Caspi et al 2000). Nevertheless, in cases with high suspicion of malignancy, laparotomy is often the first choice to avoid tumour seeding along port tracts. However, even at open operation, it may be difficult to distinguish between the two diagnoses as the macroscopic appearance of pelvic tuberculosis can be similar to the carcinomatosis of extraovarian carcinoma (Straughn and Robertson,, 2000).

Intraoperative frozen section of tissue specimens can be very helpful in oncological surgery (Straughn and Robertson,, 2000). Although histological demonstration of TB can be difficult, the lack of malignant cells may indicate an alternative diagnosis. This would be recommended if the resources are available.

Treatment for genital TB is medical (Global Tuberculosis Control Report 2008, Nebhani et al 2004). The national programme of TB control in Madagascar recommends a regime of eight months treatment, with quadruple therapy (rifampicin, isoniazid, ethambutol, pyrazinamide) for the first two months, followed by 6 months of isoniazid and thiacetazone.

Although most cases resolve with this regime, the long-term prognosis for patients' fertility is poor. One study estimated that pelvic TB was responsible for more than 39% of cases of tubulo-ovarian infertility (Nebhani et al 2004). Early diagnosis and the prevention of tuberculosis, including BCG immunisation campaigns, are important in order to avoid this devastating outcome.

## Conclusion

Isolated ovarian tuberculosis is rare. Its presentation can mimic that of an ovarian malignancy, including an ovarian mass, ascites and a rise in CA-125 level. It should be kept in mind as a differential diagnosis, both in developing and developed countries.
